# Adoption of Evidence-Based Health Promotion Programs: Perspectives of Early Adopters of Enhance^®^Fitness in YMCA-Affiliated Sites

**DOI:** 10.3389/fpubh.2014.00164

**Published:** 2015-04-27

**Authors:** Basia Belza, Miruna Petrescu-Prahova, Marlana Kohn, Christina E. Miyawaki, Laura Farren, Grace Kline, Ann-Hilary Heston

**Affiliations:** ^1^Health Promotion Research Center, University of Washington, Seattle, WA, USA; ^2^School of Nursing, University of Washington, Seattle, WA, USA; ^3^School of Social Work, University of Washington, Seattle, WA, USA; ^4^Healthy Living Department, YMCA of the USA, Chicago, IL, USA

**Keywords:** older adults, RE-AIM, physical activity, dissemination, adoption, evidence-based programs, community intervention, dissemination framework

## Abstract

**Purpose:**

To identify facilitators and barriers among early adopters of Enhance^®^Fitness (EF), in Young Men’s Christian Association-affiliated (Y-affiliated) sites from the perspective of program staff. EF is an evidence-based group exercise program for seniors.

**Methods:**

This qualitative study used semi-structured phone interviews with 15 staff members representing 14 Y-affiliated sites. Interviews were digitally recorded, transcribed, and analyzed using qualitative content analysis informed by the RE-AIM framework.

**Findings:**

Staff were, on average, 48.7 years old (SD 13.5) and had been involved with EF for 5.2 years (SD 3.1). Key themes related to facilitating adoption of EF were: match with the Y mission, support from different organizational levels, match between the target population need and EF, initial and on-going financial support, presence of champions, novelty of EF, an invitation to partner with a community-based organization to offer EF, and program-specific characteristics of EF. Key themes related to barriers interfering with EF adoption included competing organizational programs and space limitations, limited resources and expertise, and costs of offering the program.

**Implications:**

Our findings identify the types of organizational support needed for adoption of evidence-based health promotion programs like EF. Recommendations for practice, research, and policy based on the findings, including assessing organizational readiness, researching late adopters, and developing revenue streams, may help facilitate program adoption. Packaging and sharing these practical recommendations could help community-based agencies and nationally networked organizations facilitate adoption of EF and other evidence-based programs.

## Introduction

Since the development of evidence-based medicine (EBM) over two decades ago (1), the Centers for Disease Control and Prevention (CDC) has promoted healthy aging with an emphasis on reliable, efficient, and cost-effective care models with measurable outcomes (2). The goal of evidence-based programs is to create healthier communities and prevent chronic diseases (3). Translating evidence-based programs from research studies to community practice and sustaining them is a top priority for public health researchers and practitioners.

Evidence-based health promotion programs for older adults have been adopted and implemented by organizations in communities throughout the United States (4, 5). Enhance^®^Fitness (EF) is an evidence-based group exercise program for frail and active older adults (6–8). The one hour classes meet three-times a week and include exercises for cardiovascular endurance, balance, flexibility, and strength. Research has demonstrated that EF improves upper and lower body muscle strength and flexibility (9, 10). EF participants report improved over-all health (9). Participation in EF has been associated with healthcare costs saving. The average increase in annual total healthcare costs was less among EF participants compared to non-participating controls in a managed health care plan ($642 vs. 1175) (6). Among Medicare beneficiaries, enrollment in EF was associated with per person medical savings of $945/year after enrollment (8). Since 2001, there have been a total of 42,560 EF participants (unduplicated) served at a total of 689 sites in 33 states and Washington DC (Susan Snyder, personal communication, Senior Services, 2014 April 14).

Senior Services (Seattle, WA, USA) licenses and disseminates EF to multipurpose social service agencies, faith-based organizations, retirement communities, and recreational organizations. With more than 10,000 community locations, Young Men’s Christian Association (Y) is a leading non-profit committed to improving the nation’s health and well-being by offering programs that nurture the mind, body, and spirit. From 2005 to 2012, 116 Y-affiliated community sites adopted EF. These included both classes on-site at Y brick-and-mortar buildings, and classes licensed by Ys but offered in community settings such as churches or retirement communities. These sites represent a first stage in the adoption of EF by a nationally networked organization; as such, their experience is likely to inform efforts to scale-up adoption of EF and other evidence-based programs. In this paper, we refer to these sites as early adopters (11).

Various models have been used to evaluate evidence-based health promotion programs. One of these program planning and evaluation models is RE-AIM. RE-AIM, an acronym for Reach, Effectiveness, Adoption, Implementation, and Maintenance, is a systematic process that researchers, practitioners, and policy makers use to evaluate the dissemination of health promotion programs (12). Within the RE-AIM framework, adoption refers to the “proportion and representativeness of settings willing to initiate a given program” (13). Understanding how adoption of interventions plays out in different organizational settings is critical to the current and potential impact of an intervention (14).

Previous research has identified a number of motivating factors for program adoption: participant interest and/or demand, proven safety and effectiveness for older adults, cost, and a well-rounded program structure that attracts multiple groups (13). Additionally, the availability of resources is important for program adoption (15). Facilitating factors for program adoption identified in previous studies include having a curriculum, availability of training, space, and equipment (12), awareness of the importance of promoting physical activity in the community and internal support for physical activity interventions (15), sufficient funds, leadership support, capable staff, and successful partnerships and collaborations (16). Barriers include scheduling issues, lack of space, and insufficient participant recruitment efforts (13), program cost (16, 17), and lack of leadership, and time and training (16). These findings reinforce the idea that adoption can be improved by developing organizational support and capacity to deliver a program (18).

The research question for this study was: what are the facilitators and barriers among early adopters of EF in Y-affiliated sites from the perspective of program staff. Based on our findings, we provide practice, research, and policy recommendations that may help inform the adoption of other evidence-based programs in community settings. Increasing the number of community organizations that adopt evidence-based health promotion programs for older adults will contribute to the goal of creating healthier communities and preventing chronic disease.

## Materials and Methods

### Design

This qualitative study used semi-structured individual phone interviews with 15 staff from 14 Y-affiliated sites that had responsibility for the oversight of EF.

### Measure

We developed a structured interview guide for staff with questions that were informed by the RE-AIM framework (Table 1). We piloted our guide, which adds rigor to our data collection process (19). The interview guide contained a total of 39 questions and probes about benefits of EF, fitness checks, facilitators and barriers/challenges to offering EF, staff responsibilities, support from the Y management for offering EF, and strategies to recruit EF participants and instructors. Seven of the 39 questions included skip patterns (#5, 9, 12, 15, 16, 30, and 39). For example, item #9 asked: were you involved in the original adoption of EF? If the staff answered in the affirmative, then the follow on question was asked: if yes, how did your YMCA come about offering EF? If the staff answered not in the affirmative the interviewer immediately went onto the next question. Ten of the 39 items were close-ended or demographic items requiring short responses (#1, 2, 3, 7, 8, 17, 20, 36, 37, and 38). Additionally, we asked staff their age, duration of time involved with EF, educational level, and title of their position.

**Table 1:** **Interview guide for Y administrative staff**.

**INFORMATION ABOUT THE INTERVIEWEE AND ENHANCE^®^FITNESS OVERSIGHT**
1. What is your title with the YMCA? How long have you held this position? 2. Approximately what year did your YMCA first become involved with EF? 3. Approximately what year did you become involved with EF? 4. What are your responsibilities related to EF? 5. Are you responsible for the oversight of EF with your YMCA? If not, what is the title of the person responsible for oversight? 6. What are the responsibilities related to oversight of the operations of EF with your YMCA? 7. Is your YMCA still offering EF classes? Yes No 8. What, if any, other exercise classes and/or programs does/did your YMCA offer specifically for seniors (defined as older than 62 years) besides EF?
**ENHANCE^®^FITNESS READINESS AND ADOPTION**
9. *Were you involved in the original adoption of EF? If yes, how did your YMCA come about offering EF? 10. *What was the primary motivation for your YMCA getting involved in EF? 11. *What were some of the other motivations for your YMCA getting involved in EF? 12. *Does/did your YMCA receive funding to provide EF classes? If yes, what kind of funding? Source of funding? Use of funds? (Probe: staff support, participant fee offset, marketing, etc.) 13. Do you think your YMCA has/had the right and adequate number of … to support EF classes? Staff Instructors Class materials (chairs, weights, etc.) Space/room 14. Are there any resources you wish you had, or had more of, to support EF classes at your YMCA? 15. *Is there someone at your YMCA that is a champion for EF? (A champion can be paid staff or a volunteer who helps keep classes going, recruits new participants, or works to expand the program, for example) If YES: can you describe some of the things this person does to champion EF? 16. Are there paid and/or volunteer staff that manage or oversee EF operations with your YMCA? (This would include scheduling classes, managing instructors, and/or answering EF questions for current or potential participants.) If YES: how was staff recruited or selected to manage EF with your YMCA? 17. Is the management of EF: Centralized and occurs at the association-level, or De-centralized and managed at the branch level? 18. What are/were the methods used to recruit EF instructors to teach classes with your YMCA? 19. What are/were the methods used to recruit participants to EF classes with your YMCA? 20. Location of EF classes: Are EF classes held at: a) your YMCA, b) off-site locations, or c) both? If off-site, what type of locations? If more than one site, list all sites and be specific as to type of site. (churches, senior centers, community centers, Parks and Recreation facilities, schools, and retirement communities)? 21. What is/was the primary motivation for you personally getting involved in EF? (Probe: part of my job, personal interest, etc.) 22. What are/were some of your other motivations for you getting personally involved in EF? (Probe: saw the benefits, have aging family members that had benefited from this or similar programs.)
**EXPERIENCE WITH ENHANCE^®^FITNESS**
23. What benefits do you think your YMCA gets/got from participating in EF? (Probe: member engagement, community outreach, and serving a need). Is/was your YMCA reimbursed in any way for offering EF classes? If yes, by whom? Do/did your participants pay a fee to participate in EF classes? If yes, how much? If the fee changed over time, provide a range but note the current amount Does your Y ever offer EF: On a sliding scale? For reduced cost? How reasonable do you think this fee is? How does this fee compare to other like classes or programs? (Probe: More, same, less than?) Are EF classes through your YMCA available to both Y members and non-members? Does your Y track conversion rates of non-member program participants to members? (e.g., For 10 non-member participants, 1 becomes a member.) If YES: what is the conversion rate? Do you know if any of your EF participants are/were reimbursed for participating in EF, such as through health insurance? If yes, by what plan or program? 24. What do/did you like most about EF? (Probe: full classes, positive reports from participants, outcomes tracking) 25. What do/did you like least about EF? (Probe: time of the day the class is offered, instructor, room the class was held, conducting the fitness checks) 26. What are/were characteristics of EF classes that you think make them successful? (Probes: instructor ownership of the class, size of class, well-ventilated room, good time of day, room easily accessible, and class offered immediately before or after another event like a meal program) 27. What are/were characteristics of EF classes that you think are barriers to success (probe: too small of a room, mismatch between instructor and participant characteristics, high fees, time of day)? 28. If the class is no longer offered: why do you think EF is not/no longer offered at your YMCA? (Probe: cost of running EF, no instructor, instructor not a good match with participants, not enough participants, not large enough space, and other concurrent exercise classes) 29. What are/have been your challenges with offering EF? (Probe: finding instructors, frailty of participants, and for the participant transportation to and from class) 30. Are/were there particular issues you have faced in offering EF? If YES: can you describe these issues, and how you have handled them? 31. What does/did your YMCA do that you think makes EF appealing to: (Probes: advertising, offering at prime time, and offer it at no or low cost) Participants and members at your YMCA? The greater community that your YMCA serves? If your Y receives funding to offer EF classes (separate from member due or class fees), who are the other funders (probe: specific organization and individual)? 32. What does/did your YMCA do that you think makes EF not appealing? 33. What are the reasons you believe your YMCA has/not been able to implement/maintain EF classes? 34. Do you have any recommendations for changes to EF based on your experiences? 35. How was your experience with EF been similar to or different from other classes at the YMCA, particularly fitness classes or classes for seniors? 36. Regarding your Y members: About how many members does your YMCA serve? About what percent of your members are 65 and older? About what percent of your members are in the 50–64-year-old age range?
**DEMOGRAPHIC ITEMS**
37. What is your age? 38. In what education category do you fall: less than college degree, some college or college degree, and more than college degree?
**CLOSING**
39. Is there anything else you would like us to know about your experience with EF with your YMCA?

*Items with high relevance to the findings presented in this paper are noted with an asterisk*.

### Procedures

Our study was determined to be exempt by the University of Washington Institutional Review Board. We obtained administrative program records from Senior Services of all Y-affiliated program sites that had offered EF between January 2005 and June 2012. Inclusion criteria for this study were EF program management staff listed in the administrative program records from 2005 to 2012 in 116 Y-affiliated sites and who had complete contact information. Exclusion criteria were: (1) staff on this list without complete contact information, and (2) staff during the pre-enrollment screening call were determined that they did not have experience with EF. The list included 94 names of EF program management staff; of which 75 has complete contact information. Recruiting letters were sent to those 75 staff. Additionally, a Y-USA staff member (AHH) sent emails to the staff employed by Ys inviting them to participate in our study. Two reminder recruiting postcards were sent to staff who had not responded to the initial recruiting letter; reminders were sent at 2 and 4 weeks after the initial mailing. Interested staff called a study phone line or sent an email to the study email account. Twenty-five out of 75 (33%) responded to the recruiting letter, and 8 were determined ineligible for the study. Two staff were placed on a wait list. The study recruiting coordinator (LF) tracked and responded to all phone and email messages, determined eligibility, and scheduled interviews. At the beginning of each phone interview, verbal informed consent was obtained. Interviews were conducted by two members of the research team (BB and MPP). Each study participant received a gift card for $20. Interviews ranged from 30 to 71 min (average duration was 47 min) and were digitally recorded. In 2008, Gill et al. (20) note that when conducting interviews the length varies depending on the topic, researcher and participant. However, on average, the duration of interviews about health care topics is 20–60 min (20). The average length of our interview is within this range.

The interviews were transcribed by a professional transcriptionist using a “lightly edited verbatim” style for readability with an emphasis on sentence structure. This is a more frequently used style over “strictly verbatim” since it is executed without compromising the actual content or altering the intended expression (21). “Lightly edited” is simply used to refer to the reduction of superfluous words such as “hmm, ah, mm, you know, well, yeah, uh-huh.” Transcripts are still considered full and complete and do not in any way deviate from representing the full intent and thoughts as expressed by each individual respondent. Transcripts were then entered and analyzed in ATLAS.ti version 7.

### Data analysis

Our research team had content expertise in dissemination and implementation science, administrative management, community-based participatory research, gerontology, public health, and healthy aging. All members of our team that were involved in the analysis had previous experience in conducting qualitative analysis. Our study used qualitative content analysis (22) to identify facilitators and barriers to the adoption of EF. A codebook was developed using a combination of *a priori* themes addressed in the interview guide and additional themes identified through the initial review of interview transcripts. The team met weekly for 3 months to discuss and come to agreement on coding rules. A dyad (BB and GK) double-coded a subset of five transcripts until agreement reached 85%. Remaining transcripts were divided between both team members (BB and GK) and coded independently. After the initial coding, we used a deductive approach (23) to review the coded text within the RE-AIM framework component of adoption (24). Descriptive statistics were calculated for demographic items including staff age, duration of involvement with EF, and education level.

## Results

Study participants were, on average, 48.7 years old (SD 13.5) and had been involved with EF for 5.2 years (SD 3.1). Three staff (20%) had some college education, seven (47%) had a college degree, and five (33%) had more than a college degree. Ten staff members (67%) were employed by a Y while the remaining five staff (33%) were employed by other organizations (faith-based organization, senior center, social service organization, and residential facility). Titles and levels of responsibility varied: five were Health and Wellness staff, four had roles specific to older adults, six were at the program coordinator/instructor level, and eight were at the program manager/director level or above.

Staff interviewed represented 10 different YMCA associations in six states. Fourteen out of 15 staff worked in locations where classes were conducted; one staff worked in an administrative office. Ten sites currently offered EF classes; the remaining five had previously offered EF but did not have active classes at the time of the interview.

In the remainder of this paper, we summarize our findings regarding facilitators for adoption of EF and barriers that interfered with adoption of EF, with representative quotes presented in Tables 2 and 3, respectively.

**Table 2:** **Facilitator themes for adoption of Enhance^®^Fitness (EF): perceptions of Y staff**.

	Theme	Transcripts represented	Exemplar quotations
1	EF matches well with Y mission	8 of 15 (53%)	Adopting an evidence-based program fit well with the Y goals and standards and was congruent with the Y mission. The Y mission includes providing programing that helps to improve the health and fitness of older adults (Age 30, 11 years with the Y).
			It’s back to the spirit of mind and body of what the YMCA does (Age 33, 11 years with the Y).
			We knew it was an evidence-based program, one that fit well with the Y goals and standards (Age 30, 11 years with the Y).
			I think it falls into our focus areas i.e., healthy living for seniors and social responsibility as well. We’re being responsible when we provide those types of exercise programs (Age 29, 5 years with the Y).
2	Organizational support	5 of 15 (33%)	The association office asked us if we would be interested. Because of the clientele we have, we have lots of seniors, of course I stepped up to the plate and said: “Yes, definitely let’s try this for our seniors.” That’s how we got involved (Age 41, 14 years with the Y).
			I think having a focus and support from junior management is important (Age 38, 8 years with the Y).
			I think you need to have an Executive Director or CEO really understanding what it means to deliver evidence-based programs (Age 38, 8 years with the Y).
			I think it’s always good thing to bring something new in. It was driven by the Y of the USA. And then also I was asked to do this by our Health and Wellness director of the main branch (Age 51, 15 years with the Y).
3	Match with the target population	10 of 15 (67%)	We had been looking for an older adult program because we have a large aging population in our community. It has been an age bracket that had been underserved at our Y (Age 30, 11 years with the Y).
			We are known for the fact that we offer programing that is valuable to the community and to the seniors in the community. EF is one of those programs (Age 63, 2 years with Y-affiliated site).
			We are in close proximity to (low income housing) and so this is a very easy place for them to come. It’s convenient for them. If we’re talking about people that are low income and don’t have money for public transportation, it makes it very easy for them to do something to take care of their healthy living (Age 63, 2 years with Y-affiliated site).
			It is completely appropriate for many health seekers and people who struggle with becoming more active or staying active (Age 38, 8 years with the Y).
4	Financial support	5 of 15 (33%)	When we started it, we started with the [state department of health]. … they gave us a grant basically along with other YMCAs in [the state] with all of those being downstate. They basically paid for my staff’s training and they sent us. I think that they also paid for all of our equipment. They were a huge, huge partner in this and for us being able to start EF when we did (Age 33, 11 years with the Y).
			The [state] contacted us and we’ve been working with them for some other programs. They offered to help with the initial training, and that’s where we learned about the program (Age 30, 11 years with the Y).
			We offer financial assistance. Based upon income I can give participants a certain percentage off the price of the class. And then based upon some of the grants that we have been given, I can give them even a higher percentage off. We do the best that we can to really make it happen for them. I don’t like saying no to anybody (Age 29, 5 years with the Y).
5	Champions	10 of 15 (67%)	I am a go-getter and if I hear something, I go after it because it is beneficial to our residents (Age 65, 6 years with the Y-affiliated site).
			For me, personally, it was something else for me to offer to the seniors. I absolutely love working with the active older adults (Age 41, 14 years with the Y).
			The more and more I learned about it, the more I loved it. I didn’t really know of any other like evidence-based programs for older adults. I really liked the pre and post-tests that they did. It just seemed like a great program (Age 33, 11 years with the Y).
			I told my boss about it and how I thought it would be beneficial. I told our members about it because I wanted to get them on board and get them excited. I did anything I could when the (grant sponsor) people came over. I did everything I could to promote our space (Age 60, 7 years with Y-affiliated site).
6	Novelty of EF	5 of 15 (33%)	I thought it would be something different, you know? I thought it would be more different and something that we could offer to our seniors (Age 41, 14 years with the Y).
			It was just something new and exciting, evidence-based. It was everything we wanted (Age 51, 11 years with the Y).
			I think it’s always good thing to bring something new in (Age 51, 16 years with the Y).
			I just wanted to have a varied program offering, and I thought this would fit…I wanted to keep the people who come here happy with our center. I want to give them a variety of things, and so I don’t want anything to be stagnant (Age 60, 7 years with Y-affiliated site).
7	Invitation to partner with another organization to offer EF	8 of 15 (53%)	I think that I thought it looked like a great program. Our partnership at the [state department of health] was so strong. They really wanted to help the YMCAs start it (Age 33, 11 years with the Y).
			Sometimes the senior centers request us to do a program. That is kind of how it happened. It was just really good timing when we started EF because they were requesting that we come and do some different things. We thought it would be perfect and so it just kind of fell into place (Age 33, 11 years with the Y).
			I was working with a grant writer at [a university]. I was looking for something that we could get through a grant. This is the something she came up with (Age 60, 7 years with Y-affiliated site).
			Someone [YMCA staff member] called [my manager] and said: “We have this EF class and would you want to be our pilot program?” And she said “Absolutely, yes! That’s how it all started” (Age 51, 11 years with the Y).
8	Program-specific characteristics of EF such as being evidence-based and with name recognition	10 of 15 (67%)	It is evidence-based and has got solid backing. It has a proven track record and can meet the needs that are out there (Age 30, 11 years with the Y).
			I won’t touch anything that does not have data or an evidence-based curriculum, especially as related to chronic disease management (Age 38, 8 years with the Y).
			It is an incentive to bring people in when they know that you have a program that is known throughout the country. It’s a recognizable name. You are branded already (Age 63, 2 years with Y-affiliated site).

**Table 3:** **Barrier themes to adoption of Enhance^®^Fitness (EF): perceptions by Y staff**.

	Theme	Transcripts represented	Supporting quotations
1	Competing programing	5 of 15 (33%)	For group classes we have dance, water aerobics, step aerobics, spinning, and the range of movement class from [another exercise program]. We have additional programs that are available at a cost, and those include our nutritional services; the EF classes; swimming lessons; different sports programs, and then small group training types of classes (Age 30, 11 years with the Y).
			We acknowledge that space is an issue … They [wellness directors] see it as oh we already have [another program], our program for active older adults. Why would we want to do this one? (Age 38, 8 years with the Y).
			… We [offer EF] off-site. We are not in our own building anymore. It was to save on rent … The big room is often taken up with children’s camps and things like that (Age 60, 7 years with Y-affiliated site).
2	Limited resources and expertise	7 of 15 (47%)	The staff did not see the benefit or the value to their people (Age 45, 10 years).
			… And getting our health and wellness directors to understand and not condemn it, like “What’s in it for us?” (Age 38, 8 years with the Y).
			… Where are we going to put it; who is the instructor going to be; who’s going to pay for this, or where are the funds coming from (Age 51, 16 years with the Y).
			I know the whole issue is that people don’t have time. There is a lack of staff. We have it here, too, and so I know some of the issues (Age 65, 6 years with Y-affiliated site).

### Facilitators for adoption of EF

Key facilitators identified by staff, which contributed to EF adoption, were match with the Y mission, support from different organizational levels, match between the target population need and EF, initial and on-going financial support, presence of champions, novelty of EF, an invitation to partner with a community-based organization to offer EF, and program-specific characteristics of EF, such as being evidence-based and having a recognizable name.

#### Match with the Y mission

The Y mission is to put Christian principles into practice through programs that build a healthy spirit, mind, and body for all (25). Staff employed by Ys and community organizations with whom the Y partners noted that EF’s evidence-based curriculum was a good match with the Y mission since it is proven to improve older adults’ physical health in a fun and engaging atmosphere that promotes social interaction. Staff expressed strong commitment to the Y mission and to addressing unmet demographic needs: “Adopting an evidence-based program fit well with the Y goals and standards and was congruent with the Y mission.” The staff saw it as their responsibility to keep seniors socially and mentally involved so they were not isolated at home. Another staff member mentioned, “Our strategic initiatives and our strategies roadmap for our association states very clearly that we will have a growing focus on expanding our senior membership and increasing our programing to meet the needs of the aging population.” The staff were committed to providing programs that seniors enjoyed and needed. The Ys’ values promote inclusiveness, which is operationalized by staff providing programing to improve the health and well-being of older adults.

#### Organizational support

Support from different organizational levels of the Y also served to facilitate adoption. For example, staff were consulted when EF marketing materials were developed. This helped the staff feel like they had a say in how and to whom the program was being promoted. Staff also felt that adoption of EF was facilitated with support from junior management, Wellness Directors, Executive Directors, and CEOs.

#### Match with the target population

Enhance^®^Fitness was more easily adopted when the staff perceived there was a good fit between the needs of the target population (older adults) and the program itself (EF). Older adults were considered to be in an age bracket that had previously been underserved by the Y. A perceived gap in programing led staff to look for exercise programs like EF because it was a valuable addition for the senior community. Because EF exercises can be adapted to suit participant abilities, the staff felt it was a very inclusive program in which older adults at varying levels of function could participate. In addition, some of the EF classes were offered off-site in settings that catered to older adults and with which the Y branch had been partnering. One such setting was a retirement community. One staff commented: “the residents [in assisted living] were good candidates because they were at a point where they had not been exercising. We could start at the beginning and see where their progress was which would not have been if we brought it into our Y and tried to offer it to our regular seniors”.

#### Financial support

Initial financial support was an important factor in the adoption of EF. Several staff members reported receiving funding through grants at the time of EF adoption to cover EF training for their instructors, weights and other equipment, licensing fees, and/or instructor salaries. These funds also allowed Ys to offer the program at no charge to participants in some sites.

#### Champions

Champions for both EF and older adult programing facilitated adoption of EF. When asked to identify champions for EF, staff identified both paid staff and volunteers who fully embraced EF, and passionately and frequently promoted the program both within and outside the Y. Staff champions advocated for and secured resources to launch the EF program. Champions described themselves as “go-getters,” extolled the virtues of EF, and communicated often with managers, staff, and site members about the benefits of EF. One staff champion expressed adopting the “we will make it work” attitude when it came to rolling out EF for the first time. Volunteer champions welcomed new comers, brought guests to class, and took on other tasks such as setting up fitness check areas. “[Champions] just do it on their own. Nobody asked them to do that (in reference to setting up fitness check areas). They just love the community that EF provides and obviously the physical benefits. They want to capture anybody that comes into class and really helps them feel that same way.”

#### Novelty

Staff were looking for new and exciting programs to offer older adults. They viewed adopting EF as an opportunity to keep their programing fresh, and valued being an early adopter when the program was just getting started: “Back then [when EF was adopted] EF was kind of an experiment. There were only a few sites in the country offering it, I believe, and so I thought that it would be nice to be part of that group.”

#### Invitation to partner

The initial adoption of EF by Y-affiliated sites was often triggered by an invitation from an established community partner, providing motivation to adopt EF. The Y has close links with community partners and values their suggestions. Being part of an active community-based network that also provides services to older adults positions the Y to be on the cutting-edge of learning when new programs are launched. Established relationships with state departments of health and affiliations with academic and philanthropic organizations were often key to adoption. These relationships afforded access to financial resources that provided initial start-up and on-going funding for EF. One staff member noted: “the opportunity to work with and partner with an outside agency to help address another portion of our population definitely interested me.” There were also examples of invitations to partner with new organizations, such as assisted living communities that had never had a program like EF. An invitation from a new partner “opened doors.”

#### Program-specific facilitators

There were program-specific facilitators that helped with adoption. Staff described EF as being unlike other programs they had offered. Most frequently mentioned was EF being evidence-based, branded, and having name recognition. Staff reported that EF was, “… an easy sale as it was proven to improve things,” and “it has solid backing.”

### Barriers to the adoption of Enhance^®^Fitness

A number of factors that interfered with the adoption of EF were noted such as competing senior programs and space limitations, limited staff resources, and costs of the program.

#### Competing programing and space limitations

Staff noted one of the barriers to offering EF was that the Y Association was already offering a number of other programs for active older adults, and the health and wellness staff did not see the need or benefit to offering another one. Additionally, in some Y sites staff reported there was a “space crunch” with rooms that would be appropriate for offering EF being allocated to other programs such as camps for children. One staff member said: “we no longer had the luxury of having two senior programs running because of space limitations.” One staff that was trained to offer EF but never did said: “we were having a time and space crunch. It wasn’t anything wrong with the program *per se* but we’re not going to take away our already very strong programs and try something new.” Off-site locations like retirement communities also had issues with finding adequate space.

#### Limited resources, expertise, and program costs

Another factor that interfered with the adoption of EF was the lack of staff resources, both in terms of time availability and the need to find instructors with appropriate skills for working with older adults. Staff noted that costs of the program were a potential barrier to adoption: rent, materials, and instructor costs, on the one hand, and affordability for participants, on the other, were taken into account before deciding to adopt EF.

## Discussion

In this study, we examined the facilitators and barriers to the adoption on EF in early adopter Y-affiliated sites. The Health Promotion Research Center (HPRC) dissemination framework provides a context for interpreting the findings from this study and informs the translation of our findings to other community-delivered, evidence-based programs (26). The HPRC dissemination framework incorporates the terminology of the RE-AIM framework such that the definition of adoption is consistent between the two frameworks.

**Figure 1:**
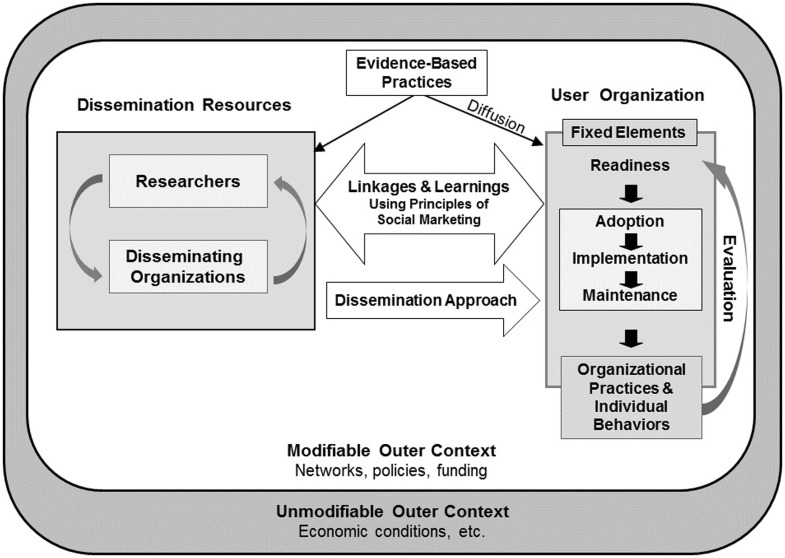
**The HPRC dissemination framework [taken from Harris et al. (26)]**.

The HPRC dissemination framework (Figure 1) identifies three main actors involved in the dissemination of an evidence-based program: researchers, disseminating organizations, and user organizations. Researchers and disseminating organizations partner to develop a dissemination approach that is suitable for the targeted user organizations. The approach is built on learnings about the user organization’s characteristics and readiness for adoption and implementation, and is continually refined through the collaboration of the three main actors. At the same time, all actors operate within a broader context that includes both modifiable and unmodifiable components, such as funding and partnerships, and economic conditions, respectively.

Study participants identified several facilitators and barriers related to the characteristics of Y-affiliated sites, the user organizations in this study. Ys have made major strides in recent years to become leaders in community-based health promotion programs. It was readily apparent that there was a strong fit between EF and the Y mission to offer older adults an environment that promotes physical and emotional health, and the availability of resources to ensure adequate programing. This rich environment also included champions for both EF and older adult programs. Programs with this type of fit have an enhanced ability to sustain themselves. On the other hand, our findings suggest a need for improved evaluation of organizational readiness prior to the adoption of any evidence-based program. Structured and rigorous determination of organizational readiness would help organizations avoid known barriers to successful implementation, such as competing programs, and limited space and resources. Organizational readiness for change is an important precursor to the successful implementation of health promotion programs (27).

Our study also identified facilitators related to the modifiable context in which user organizations operate. Staff noted the significant role grant funding played in the initial adoption of the program, as well as the importance of building partnerships at the local and state level.

Finally, we would like to highlight the role of the disseminating organization, Y-USA, the national office of the Y. Between 2007 and 2011, Y-USA significantly increased its national effort, Activate America^®^, to engage Ys in organizational and community change focused on supporting health seekers, those who struggle to adopt and maintain a healthy lifestyle. Nearly two-thirds of Y Associations committed to Activate America and built their capacity to better align their programs, practices, and policies with the needs of health seekers. This was the Y climate when EF was being brought into the organization by early adopters. The organization was primed for embracing evidence-based health promotion programs and working with a community of adults with chronic conditions. At this time, chronic disease prevention programs were introduced as well, including LIVE**STRONG** at the YMCA, a cancer survivorship program, and the YMCA’s Diabetes Prevention Program (DPP).

Ackermann and colleagues tested offering DPP at Y sites (11, 28–30). Ys were able to increase the number of participants and offer DPP at a lower cost compared to other community settings, making Ys an ideal community partner. They noted a variety of factors contributing to the success of DPP offered at Ys: the Y is a nationwide, community-based organization reaching diverse U.S. communities; it has a successful history of adopting and implementing health promotion program for all age groups; it takes a group-delivery approach, which uses minimal over-all personnel cost; and it has a national policy to accept all participants regardless of their ability to pay the membership fee. Y-USA and its associations and branches nationwide are well-positioned to successfully adopt new evidence-based programs, and more broadly disseminate existing programs based on the program experience noted above, and the organizational infrastructure already in place.

There were several limitations in this study. First, we used a convenience sample. Convenience samples can introduce response bias, with those having positive experiences being more likely to participate than those with less favorable experience. However, staff who volunteered to participate in this study shared a range of experiences, both positive and negative, related to the adoption of EF. Second, our sample size of 15 may have limited our ability to reach conceptual saturation with regard to the research questions. After completing 15 interviews, the research team determined that additional interviews were unlikely to return substantively new information, and that we had reached conceptual saturation. Third, inherent in qualitative methodologies is the potential issue of transferability. To minimize issues with transferability and assure our findings would have applicability in other contexts, a partner from the Y (AHH) was actively involved in all phases of our study and another Y staff member served on our Project Advisory Group. Fourth, some of the staff had started overseeing EF up to 8 years prior to the phone interviews, resulting in potential recall bias with respect to the initial decisions and process of adopting EF. Last, Y staff who were the target of our interviews were in positions in which they had responsibilities for program management and also could adopt new initiatives like EF. When further exploring the concept of adoption, it would be valuable to also include senior leadership who are likely to be key decision makers and have an influence in whether a new program would be considered, paid for and/or adopted.

Based on the findings in this study, we propose practice, research, and policy recommendations for the adoption of evidence-based health promotion programs by community organizations. They are summarized in Table 4, which also includes the facilitator/barrier themes they address.

**Table 4:** **Recommendations for practice, research, and policy in the adoption of evidence-based health promotion programs**.

	Facilitators addressed	Barriers addressed
**Recommendations for practice**
Assess fit with the organizational mission	Match with mission	
Assess fit with other programing	Novelty, match with target population	Competing programing
Identify existing community partners and new potential partners	Invitation to partner, financial support	Limited resources and expertise
Identify capable staff and instructors	Champions	Limited resources and expertise
Identify training and technical assistance for staff and potential instructors		Limited resources and expertise
Assess cultural and demographic needs of the target population	Match with target population, match with mission	
Assess physical space and time constraints		Limited resources and expertise
Assess start-up and on-going costs and offsetting funding/revenue	Financial support	Limited resources and expertise
**Recommendations for research**
Explore adoption among majority and laggard adopters, and compare to early adopters		
Explore influence of adoption on implementation and maintenance		
**Recommendations for policy**
Explore policy approaches to revenue development	Financial support	Limited resources and expertise

Our practice recommendations focus on organizational readiness for adopting new programs. Assessing organizational readiness, formally or informally, identifying gaps in readiness, and addressing any gaps may improve adoption (11). Organizational readiness can be evaluated through the following activities: assessing fit of the program with organizational mission; assessing overlap with other programing, and physical space and time constraints; identifying potential partners and funding/revenue models; identifying capable instructors and securing training and technical assistance for staff and potential instructors; and assessing cultural and demographic needs of the target population. Addressing these aspects of readiness may provide opportunities to open dialog with partners and stakeholders, or develop supporting resources like a business plan to support successful adoption.

The practice recommendations outlined above are based on the experience of early adopters. However, the adoption experience of majority adopters and late adopters/laggards may face different facilitators and barriers (11). Future research is needed to better understand the spectrum of adoption in all phases, and how early adopters’ experience may influence later adopters. In addition, successful adoption should lead to program implementation and, ultimately, maintenance. Additional research on how adoption strategies influence subsequent implementation and maintenance of evidenced-based health promotion programs could contribute to development of best practices for the translation of research into practice.

Finally, broad policy support for evidence-based programs may create an environment more primed for successful adoption. Policy support may include establishing a revenue stream to offset program costs, an approach that has been seen in other programs (30, 31). Policy approaches to developing revenue streams may be fruitful among evidence-based programs demonstrating health improvements and reductions in health care costs (6, 8).

## Conclusion

While these recommendations are based on our study of the experience of early adopters of EF at Y-affiliated sites, they are likely applicable to other evidence-based programs conducted in community settings. Facilitators and barriers to adoption apply across programs and settings (8). Furthermore, facilitators, barriers, and recommendations address modifiable aspects of adoption that may improve success, including support of the organizational mission, available resources, and options for offsetting costs. Y-USA has successfully adopted a variety of evidence-based programs, and can serve as a model for other regionally and nationally networked community organizations. Organizations looking to adopt new programs may increase their likelihood of success by applying the recommendations appropriate to their organization and program.

## Conflict of Interest Statement

The authors declare that the research was conducted in the absence of any commercial or financial relationships that could be construed as a potential conflict of interest.

This paper is included in the Research Topic, “Evidence-Based Programming for Older Adults.” This Research Topic received partial funding from multiple government and private organizations/agencies; however, the views, findings, and conclusions in these articles are those of the authors and do not necessarily represent the official position of these organizations/agencies. All papers published in the Research Topic received peer review from members of the Frontiers in Public Health (Public Health Education and Promotion section) panel of Review Editors. Because this Research Topic represents work closely associated with a nationwide evidence-based movement in the US, many of the authors and/or Review Editors may have worked together previously in some fashion. Review Editors were purposively selected based on their expertise with evaluation and/or evidence-based programming for older adults. Review Editors were independent of named authors on any given article published in this volume.
